# Characterizing Branching Vascular Network Morphology in Polypoidal Choroidal Vasculopathy by Optical Coherence Tomography Angiography

**DOI:** 10.1038/s41598-018-37384-y

**Published:** 2019-01-24

**Authors:** Chu-Hsuan Huang, Po-Ting Yeh, Yi-Ting Hsieh, Tzyy-Chang Ho, Chung-May Yang, Chang-Hao Yang

**Affiliations:** 1 Department of Ophthalmology, National Taiwan University Hospital, Taipei, Taiwan; 2 Department of Ophthalmology, National Taiwan University College of Medicine, Taipei, Taiwan

## Abstract

This study analyze the morphological characteristics of branching vascular networks (BVN) in polypoidal choroidal vasculopathy (PCV) using optical coherence tomography angiography (OCTA), and correlate imaging characteristics with clinical presentations. We presented a retrospective observational case series for fifty cases of PCV confirmed by indocyanine green angiography. Macular OCTA were done by the AngioVue. The PCV cases were classified by distinct morphologic patterns of BVN by two retina specialists and clinical features were analyzed. The sensitivity of polyp detection by OCTA was 86% after manual segmentation and that of BVN was 90%. Three distinct morphologic patterns of BVN were identified. (1) The “Trunk” pattern (47%) exhibited major vessel trunk with features including presence of drusens, thin choroid, and larger BVN area. (2) The “Glomeruli” pattern (33%) showed anastomotic vascular network without major trunk. (3) The “Stick” pattern (20%) had localized BVN and the thickest choroid. Subtypes 2 and 3 held higher recurrence rate. In conclusions, the precise visualization of BVN on OCTA supported that OCTA might be a noninvasive tool to study the morphology of BVN in PCV, which exhibits three different morphological types. Identifying the morphology of BVN has the potential to prognosticate outcomes in PCV patients.

## Introduction

Polypoidal choroidal vasculopathy (PCV) was firstly described by Yanuzzi in 1982, and was considered as a part of the spectrum of neovascular age-related macular degeneration (AMD) because of similarities in the vascular characteristics^1^. The prevalence of PCV in presumed AMD patients differs between geological regions, ranging from less than 10% in the western society to about half in Asia countries, and the disease presentation among patients is also widely variable^2–4^.

The current diagnosis of PCV mainly relies on indocyanine green angiography (ICGA), which reveals polypoidal vascular lesions and the branching vascular networks (BVN)^5,6^. However, the two-dimensional ICGA lacks the ability to localize the lesions in the different layers of the retinal and choroid as seen on optical coherent tomography (OCT)^7,8^. High-penetration and spectral-domain OCT can also provide detailed information on choroidal layers^9^. Also, ICGA is an invasive procedure which requires dye injection with risk of allergic reaction^10^, and it is highly time-consuming and unlikely to be perform at every post-treatment follow-up. With the new developments in imaging technology, OCT angiography (OCTA) provides access to visualize blood flow in the retina and choroid in a non-invasive manner^11,12^. Therefore, OCTA may be used to localize PCV and BVN lesions and abnormal vasculature, and to monitor the temporal changes of the lesions after treatment.

Moreover, the presentation of PCV varied widely among patients. Yuzawa *et al*. classified PCV into two types according to the presence of feeder vessels or not^13^. Tan *et al*. differentiated PCV into 3 subtypes according to the presence of leakage in ICGA^14^. Recently, Freund *et al*. proposed that PCV may develop from long-standing Type 1 choroidal neovascularization, in line with the believe that PCV is a pachychroidal spectrum disease^15^. However, the possible diversity of PCV presentation remains unclear. The high resolution and detection rate of BVN by OCTA may provide more detailed clues in the presentation and pathogenesis of PCV.

The purpose of the present study was to categorize and describe the morphological characteristics of BVN in PCV by OCTA, and correlated the imaging characteristics with the clinical presentations.

## Method

The patients who were diagnosed as PCV by ICGA and had received OCTA simultaneously, from August 2015 to December 2016 at National Taiwan University Hospital (NTUH) were enrolled, and the chart was retrospectively reviewed. For each case, the diagnosis of PCV was independently confirmed by two retinal specialists based on the detection of polyps with early dye pooling in ICGA. Patients were excluded if they had polyps beyond a 3 × 3 mm area from central macula to ensure high image resolution. Patients were also excluded if they had been previously diagnosed with proliferative diabetic retinopathy, myopic maculopathy, or other causes of fundus neovascularization. The study has been approved by the institutional review board of NTUH and informed consent was obtained from all participants. All investigations followed the tenets of the Declaration of Helsinki.

The demographic and ophthalmological data at the first visit, including age, sex, best-corrected visual acuity (BCVA), previous ocular treatment history, and subfoveal choroidal thickness (SFCT) were recorded. Patients with confirmed diagnosis of PCV all received the combined therapy of photodynamic therapy (PDT) and at least two injections of Anti-VEGF agents (Bevacizumab or Ranibizumab). Post-treatment BCVA in logarithm of the minimum angle of resolution (LogMAR) was recorded at 6 month after PDT. The resolution and recurrence of subretinal fluid (SRF) were also documented.

**Figure 1:**
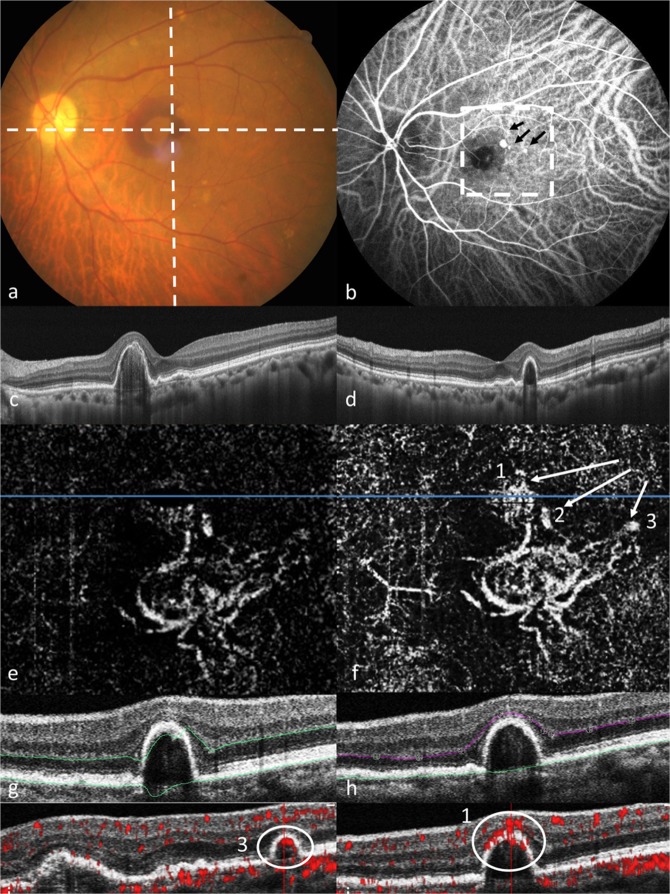
The multimodal image revealed compatible multiple polyps in manual-segmented OCTA and ICGA study and the BVN was clearer in OCTA. This was a 65 year-old female who was newly diagnosed as PCV by ICGA. (**a**) The fundus revealed nodular lesion and retinal hemorrhage. (**b**) The mid-phase ICGA revealed 3 polyps indicated by black arrows and BVN. (**c**) Transverse OCT B-scan image at the level corresponded to horizontal dash line in (**a**), revealed RPE detachment and sub-RPE iso-reflective lesion. (**d**) Vertical OCT B-scan image at the level corresponded to vertical dash line in (**a**), also revealed RPE detachment and sub-RPE iso-reflective lesion. (**e**) Automated OCTA en face image, which revealed BVN but no definite polyp. (**f**) OCTA en face image after manual adjustment of level of outer plexiform layer and bruch’s membrane, which clearly revealed 3 polyps (1~3) compatible with ICGA. (**g**) In the representative B-scan At the level of blue line in (**e**), the automatically set OPL (upper green line) and bruch’s membrane (lower green line) were deviated due to high PED. (**h**) In the representative B scan at the level of blue line in (**f**), the manually adjusted OPL (purple line) and bruch’s membrane (green line) produced en face image as in (**f**). (**i**,**j**). Polyp structure with high flow signal in angio-flowgram which lied around RPE (circle) and localization of polyp 3 (**i**) and polyp 1 (**j**).

OCTA was performed by the RTVue XR Avanti with AngioVue OCTA system (Optovue Inc., Fremont, CA, USA), which applied 2 consecutive B-scan composed of 304 × 304 of cross A-scan in approximate 2.6 seconds (70,000 A-scans/sec) to acquire angiography from OCT image by the split-spectrum amplitude de-correlation angiography software algorithm. The automated segmentation report was used for polyps and BVN detection at first, and then manual segmentation was performed. The inner and outer levels of segmentation lines were manually adjusted to the level of outer plexiform layer and Bruch’s membrane, respectively, for the enhancement of detection ability of abnormal blood flow in the outer retina. The localization and outline of polyps and BVN were achieved under the assistance of focal red signal spots in angio-flowgrams provided in OCTA system. Fig. 1 was a case example for manual segmentation adjustment.

The cases without visualization of BVN in OCTA were excluded from the morphological analysis of BVN. The demographic data, ICGA findings, OCTA detection rate, lesion area with flow signal (acquired from the AngioVue OCTA system), central retinal thickness (CRT), and treatment response (including BCVA and SRF recurrence at 6 months after therapy) were collected. The morphology of BVN in the acquired images under manual segmentation were reviewed and similarity of different cases were gathered, which then produced 3 different features of BVN appearance to prompt the classifications. The images and classification were examined by the other retina specialists in the same hospital for the detection of polyps and morphological classifications of BVN.

Between treatment-naïve and prior-treated group, the categorical variables were analyzed by chi-square tests or Fisher’s exact test, and the continuous variables by Student’s t-test. Among three different types of BVN morphology, the continuous variables were analyzed by the analysis of variance (ANOVA) tests and Scheffer test if significance reached, and categorical variables by the chi-square tests or Fisher’s exact tests. The statistical analysis was performed using SPSS software (V.21; SPSS, Chicago, Illinois, USA) and statistical significance was set at p < 0.05.

## Result

The demographic data of the 50 cases with PCV lesions within the macula area that were enrolled in the present study are summarized in Table 1. Among the cases, 29 were treatment-naïve, and 21 had received PDT and anti-VEGF intravitreal injections before. Overall, the patients had a mean age of 67.48 years old, and 32% were female. Mean BCVA at diagnosis was 0.773 in LogMAR. Automated detection rate of polyps on OCTA was 62%, which rose to 86% after manual segmentation. Among the 7 cases which could not be identified by manual segmentation, 3 cases had small polyps in ICGA and 4 cases had high hemorrhagic PED that resulted in poor signal. In the 3 cases with small polyps in ICGA, BVN could be identified in all 3 cases. Figure 1 demonstrated a multimodal image with clear outlined BVN and polyps. The detection rate of polyps and BVN in the prior-treated group was higher than that in treatment-naïve group, although the difference was not statistically significant (p = 0.684 and 0.066, respectively).

**Table 1:** The demographic data and the OCTA detection rates for polyps and branching vascular network (BVN) in the treatment-naïve and prior-treated groups.

	Total (n = 50)	Treatment-naïve (n = 29)	Prior-treated (n = 21)	P-value
Age (y/o)	67.5 ± 7.3	68.8 ± 7.1	68.0 ± 7.4	0.292*
Gender (Female)	32.0%	37.9%	23.8%	0.291^†^
BCVA at diagnosis (LogMAR)	0.773 ± 0.588	0.606 ± 0.609	0.831 ± 0.564	0.184*
ICGA-BVN	76.0%	72.41%	80.95%	0.485^†^
OCTA-Polyps (Automated)	62.0%	62.07%	61.90%	0.991^†^
OCTA-Polyps (Manual)	86.0%	82.76%	90.48%	0.684^†^
OCTA-BVN (Manual)	90.0%	82.76%	100%	0.066^†^

*Student’s *t*-test.

^†^Chi-square and Fisher’s exact test.

**Figure 2:**
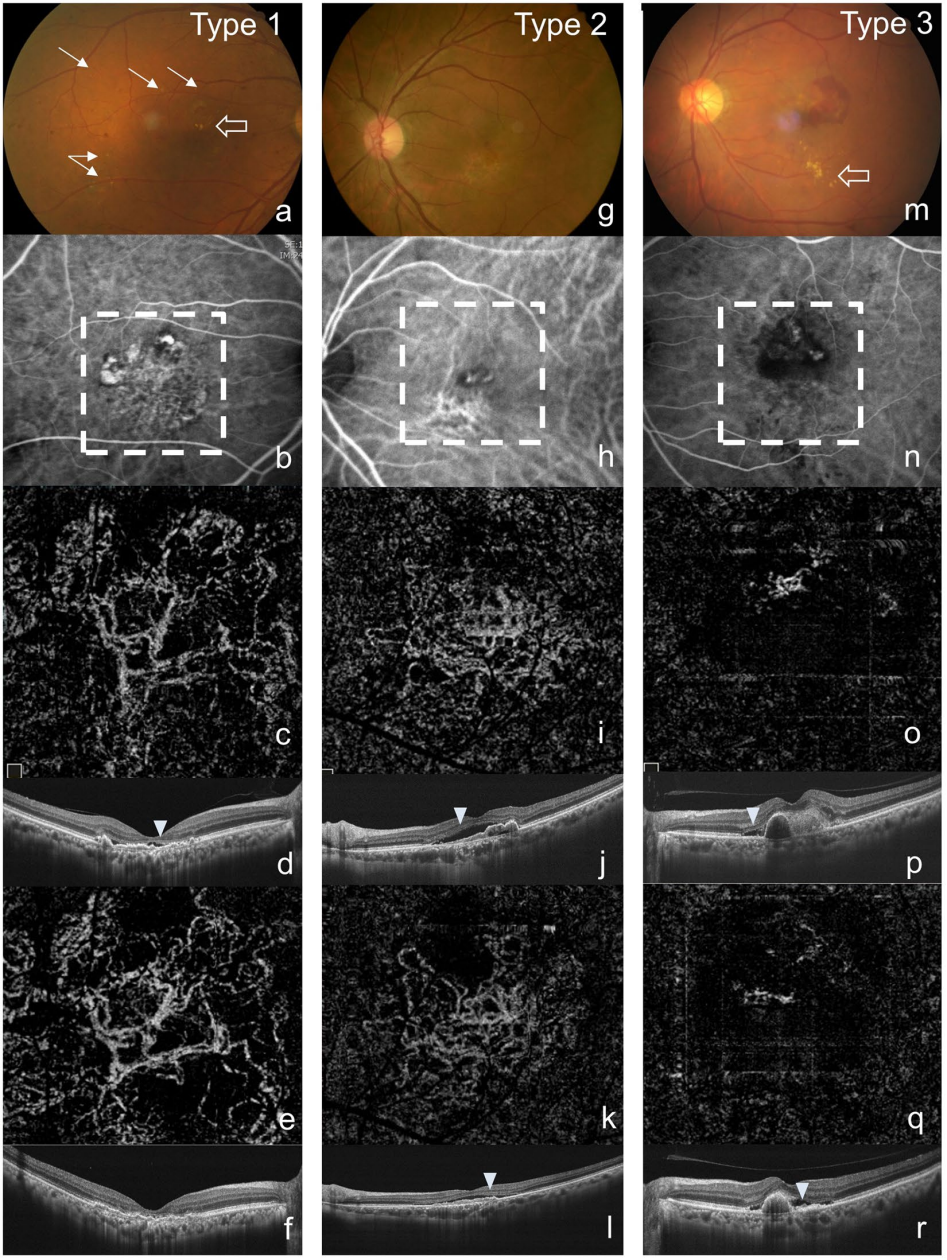
Three distinct patterns were identified from BVN on OCTA. Type 1: “The Trunk pattern”: presence of one or more main trunk of neovascular vessel and may have radiated branches pointing toward periphery of the vascular network. (**c**) The main trunk enlarged after the combination treatment while the anastomosing network shrank. (**e**) Type 2: “The Glomeruli pattern”: a vascular network with intensely interconnecting anastomosis, but without visible major trunk. (**i**) The vasculature became finer after combined therapy. (**k**) Type 3: “The Stick pattern”: localized fine neovascular network without definite pattern. (**o**) The vasculature remained after combined therapy. (**q**) The SRF recurred 6 months after treatment (arrowhead) in Type 2 and 3, but resolved in type 1 completely. There was presence of drusens in fundus photography in type 1 (arrow) and exudation in type 1 and 3 (empty arrow).

After excluding the cases without detectable BVN in OCTA, 45 cases were included in the morphological analysis for BVN. BVN morphology was categorized into three types according to distinct features on OCTA. Type 1, the “Trunk” pattern, presented with one or more main trunks of neovascular vessels and may have radiating branches pointing toward the periphery of the vascular network. Type 2, the “Glomeruli” pattern, presented with a vascular network with intensely interconnecting anastomosis but without a visible major trunk, which resembles glomeruli in nephrons. Type 3, the “Stick” pattern presented with localized fine neovascular network without identifiable feeding vessel (Fig. 2).

There were 21 cases defined as type 1 (47%), 15 cases as type 2 (33%), and 9 cases as type 3 (20%) (Table 2). The cases of Type 1 BVN exhibited larger mean lesion area which was 0.919 mm^2^ at baseline. This type of BVN always occupied the central fovea and had trend of poorer vision at diagnosis than the other two types, although the difference was not statistically significant (p = 0.117). The initial BCVA and the BCVA 6-month after treatment were 0.910 and 0.738 in LogMAR, respectively. Drusens were identified in 14 of the Type 1 cases’ (66.7%). The mean SFCT was 195.5 μm at baseline, which was thinner as compared with the other 2 types, and significant difference existed between Type 1 and Type 3 (post Hoc Scheffer test, p = 0.023). The main trunk of the BVN became more prominent after combination treatment with PDT and intravitreal anti-VEGF injections, whereas the terminal, fine vasculature shrank after treatment. The recurrence rate of SRF was 28.6% in Type 1 cases, which was significantly lower than that of the other two types (p = 0.001). However, PDT re-treatment was inevitable in every Type 1 recurrent cases due to extended lesion area and central fovea involvement.

**Table 2:** The demographic data, characteristics of ICGA and OCTA examinations, and treatment response between the 3 groups of distinct branching vascular network (BVN) morphology.

	Type 1 “Trunk” (n = 21)	Type 2 “Glomeruli” (n = 15)	Type 3 “Stick” (n = 9)	P-Value
Age (y/o)	68.0 ± 7.6	69.7 ± 7.3	65.8 ± 7.3	0.469*
Gender (Female)	52.4% (11)	13.3% (2)	22.2% (2)	0.036^†^
Treatment Naïve	52.4% (11)	60.0% (9)	55.6% (5)	0.902^†^
Presence of Drusens	66.7% (14)	33.3% (5)	22.2% (2)	0.037^†^
Submacular hemorrhage	47.6% (10)	53.3% (8)	55.6% (5)	0.903^†^
ICGA-BVN	95.2% (20)	93.3% (14)	44.4% (4)	0.001^†^
Presence of Feeder Vessel	76.2% (16)	60.0% (9)	22.2% (2)	0.022^†^
OCTA-Polyps (Manual)	90.5% (19)	100.0% (15)	77.8% (7)	0.178^†^
OCTA-SFCT (μm)	195.5 ± 66.6	239.5 ± 89.7	284.3 ± 80.1	0.019*^,‡^
OCTA-CRT (μm)	270.6 ± 60.5	282.5 ± 78.7	259.1 ± 49.8	0.691*
OCTA-BVN area (mm^2^)	0.919 ± 0.517	0.667 ± 0.381	0.404 ± 0.211	0.021*^,§^
BCVA at diagnosis (LogMAR)	0.910 ± 0.730	0.544 ± 0.357	0.530 ± 0.496	0.117*
BCVA post-PDT 6 m (LogMAR)	0.738 ± 0.656	0.476 ± 0.325	0.503 ± 0.458	0.316*
OCTA-SRF recurrence	28.6% (6)	80.0% (12)	88.9% (8)	0.001^†^
PDT Re-Treatment rate	28.6% (6)	40.0% (6)	55.6% (5)	0.368^†^

*One-way ANOVA, Scheffer test if p < 0.05, ^†^Chi-square and Fisher’s exact test. ^‡^Post Hoc: p = 0.023, between Type 1 and 3. ^§^Post Hoc: p = 0.019, between Type 1 and 3, SFCT: subfoveal choroidal thickness, SRF: subretinal fluid, CRT: central retinal thickness, PDT: photodynamic therapy.

In type 2, the mean lesion area was 0.667 mm^2^ in size, and the neovascular tissue was more or less away from central fovea, and appeared as numerous interconnecting fine vasculature. Most of the Type 2 cases occurred in male (86.7%, p = 0.036). The mean SFCT was 239.5 μm. Unlike Type 1 BVN, the diameter of the vessels generally decreased after the combination treatment with the absence of formation of main trunk. The recurrence rate of SRF was high if the BVN still exist, which was 80%. However, vision remained stable despite the high recurrence rate.

The Type 3 BVN could be easily outlined on OCTA, but was more difficult to be detected in ICGA due to its limited lesion size, with a 44.4% detection rate (p = 0.001). The mean SFCT was 284.3 μm, and 6 of the Type 3 cases (66.7%) had SFCT thicker than 300 μm, while only 1 case in the Type 1 and 2 cases in Type 2 cases each had SFCT this thick. The vessels remained existed after treatment and the SRF recurrence rate was even higher at 88.9%. The vision remained stable and was 0.503 in LogMAR for the final BCVA 6-months after PDT.

## Discussion

This present study demonstrated the application of OCTA in the diagnosis and follow-up of PCV cases. The sensitivity of polyp detection was 86% after manual segmentation, and 90% of the BVN could be outlined. We defined three different types of BVN patterns based on the morphological features on OCTA. The three types of BVN patterns not only showed different clinical presentation, but might also provide valuable information on the anatomical prognosis.

As the OCTA technology being widely applied to the detection of neovascular structures, BVN was visualized in more than 90% of the PCV cases, but the polyps were sometimes poorly identified. The detection rate of polyps by OCTA ranged from 75.2% reported by Takayama *et al*. to 92.3% in the study of Wang *et al*. with the augmentation of manual segmentation^16–19^. The authors attributed the failure of OCTA to reveal polyps to the size of polyps, the velocity of blood flow within the polyps, and the masking effect by extensive hemorrhagic retinal pigment epithelial detachment (RPED)^17,19,20^. In the present study, by manually adjusting the segmentation lines assisted by the localization of red spot on the angioflow gram, the identification rate of polyps reached as high as 86%. The limitation of polyp detection by OCTA included severe motion artifact, high PED, and small polyps, and ICGA should be performed to confirm diagnosis in clinically suspected PCV cases. The detection rate of BVN was relatively lower in Treatment Naïve group than Prior Treated group with borderline significance. There were 5 cases in Treatment Naïve group failed to detect BVN. Among them, BVN was obscured by high PED in 2 cases and RPE scar in the other 2 cases. The last case had limited area of chroidal neovascularization with SFCT exceeded 300 μm without definite branched pattern.

Due to the diverse presentation of PCV, several classification systems were introduced in the attempt to further differentiate PCV lesions into clinically relevant subtypes. Yuzawa and Kawamura had differentiated PCV into 2 types according to the presence of feeder vessels and the location of vascular network^13,21^. By their definition, the amount of the polyps, the location of BVN, and the choroid thickness all differed between the 2 subtypes. However, the precise discovery of feeder vessels requires dynamic ICGA and may be hindered by extensive hemorrhage, high RPED, or poor image quality. Inoue *et al*. classified PCV as “PCV” or “PCNV” subtypes based on the presence of typical features of pachychoroid disease^22^. The subgroup “PCV” was defined as the absence of typical AMD entities including drusen and geographic atrophy, and the presence of pachy-vessels in the choroid^22^. Similarly, Coscas *et al*. also reported 2 forms of PCV as “idiopathic PCV” and “polyps associated with neovascular AMD”^23^. The hypothesis that the etiology of polypoidal vascular lesions may result from AMD and choroidal vasculopathy respectively has raised attention, and was consistent with previous studies that discovered genetic differences that were differentially associated with typical AMD and PCV^4,24,25^. Lee *et al*. collected a large group of PCV cases and revealed the double-peak distribution of choroidal thickness in 320 eyes^26^. However, choroidal thickness is affected by age, and the cut-off point for defining “pachychroid” remains controversial, therefore making subgrouping more difficult^27^.

Since BVNs could be visualized in more than 90% of the PCV cases by OCTA, the high detection rate and detailed visualization of BVNs on OCTA examination may provide a good opportunity for morphological classification. In the study of Wang *et al*., OCTA was able to detect BVNs in 100% of cases and BVNs showed more clearly on OCTA than on ICGA^19^. They defined 3 patterns of the BVN on OCTA including seafan, medusa, and tangle, which were very similar to type I CNV in wet AMD^28^. However, the report did not disclose more detailed characteristics of these BVN patterns. In the present study, the morphology of BVN on OCTA was differentiated into 3 types.

The type 1 BVN had the “trunk” pattern and exhibited some similar entities as AMD, including the presence of drusens, thinner choroidal thickness of < 200 μm, and a larger neovascular area. The mean SFCT of the type 1 cases was close to the first peak of thinner SFCT in the study by Lee *et al*., and also the prevalence, which was 47% in this presented study^4^. Those type 1 cases also showed better success with anatomical treatment. The type 1 BVN may represent a mature choroidal neovascular membrane. Spaide pointed out the concept of an “arterioriogenesis” process of alteration in the vessel diameter in CNV network after anti-VEGF treatment, which resulted in the formation of trunk-like vasculature^29^. Similar treatment response was noted in our type 1 cases. The major trunk enlarged after the combined treatment while the anastomosing vessels shrank, possibly indicating the lower rate of SRF recurrence for less leakage from the arteriorized major trunk.

The type 3, the “stick” group, on the other side, revealed finer vessels and the smallest BVN dimension, which often presented with refractory SRF recurrence after treatment. The SFCT significantly thicker than the type 1 cases and there were 66.7% of type 3 cases that had SFCT larger than 300 um, while only 3 cases in the other two types combined. BVN was hidden in the ICG in more than half of the cases, and feeder vessel was mostly absent. Freund *et al*. described that pachychoroid-spectrum disease, especially pachychoroid neovasculopathy, manifested diffuse choroid vessel congestion, focal pigmentary epitheliopathy, and type 1 choroidal neovascularization. Polyps formation within the regional neovascular network has been reported in long standing pachychoroidal cases, which resembled our type 3 pattern^30^. Kinoshita *et al*. had found that some PCV patients show choroidal hyperpermeability in ICGA, which is also a feature of pachychoroid disease. Higher rate of recurrence in submacular fluid after anti-VEGF therapy was also found in the same study^31^. Taken together, the cases with type 3 BVN morphology in our PCV case series were more likely to be associated with pachychoroid spectrum disease.

The type 2 “glomeruli” cases, which exhibited finer anastomosing vascular network than type 1, held a significantly higher recurrence rate of SRF, and may represent a more active status. Interestingly, Kikushima *et al*. reported higher recurrence rate with older age and male gender in PCV patients, which also features type 2 BVN in this presented study^32^. Due to the limited follow-up period, whether or not the type 2 cases would follow the “arterioriogenesis” process and result in trunk vessel formation defined as type 1 is still unclear^29^. Despite the lower recurrence rate, there is a trend of poorer visual prognosis in type 1 cases and re-treatment was inevitable if recurrence happened. This consequence may be related with the larger lesion area which often involved the fovea. In contrast, there is a statistically non-significant trend of better visual acuity in the type 2 and 3 cases before treatment, because the fovea was often spared from the smaller neovascular lesion area. Cases with these patterns also demonstrated better visual prognosis after the combination treatment compared with those of type 1 with thinner choroidal thickness. Thus, choroidal thickness may be a prognostic factor in PCV cases. The choroidal thickness did rebound in recurrent PCV cases in study by Maruko *et al*., and possibly indicated disease activity^33^.

The limitation of this present study included its retrospective nature, limited case number, and short follow-up period. Only cases having polyps within a 3 × 3 mm central macular area were enrolled due to resolution consideration. Although these cases were enrolled in a single tertiary referral center, they could represent the typical PCV cases in the population. The follow-up period was only 6 months, and additional morphological changes representing different disease stages may be detected with long-term follow-up. Further studies with longer follow-up may help to clarify the role of BVN morphology in PCV patients.

In summary, we demonstrated that OCTA was suitable for the screening of polyps within central macular, showing high detection sensitivity under manual segmentation. The morphological appearance of BVN could be differentiated into 3 types. Identifying the morphology of BVN has the potential to correlate clinical presentation and prognosticate outcomes in PCV patients. We concluded that OCTA may serve as a useful tool for the detection and classification of PCV cases. The more invasive ICGA exam may be reserved for the fewer cases with negative OCTA but a high suspicion of PCV.
